# Undergraduate audiology students’ perceived competence and confidence in conducting otoscopic examination following video otoscopic training

**DOI:** 10.1186/s12909-021-02924-0

**Published:** 2021-09-27

**Authors:** Ben Sebothoma, Katijah Khoza-Shangase

**Affiliations:** Department of Speech pathology and Audiology, University of the Witwatersrand, Private Bag 3, Wits, 2050, Johannesburg, South Africa

**Keywords:** Confidence, Competence, Students, Training, Video otoscopy

## Abstract

**Background:**

Emerging research indicates that video otoscopy can be used as a teaching tool to enhance students’ ability to identify outer and middle ear pathologies. However, there is little research on the perceptions of audiology students regarding their competence and confidence following video otoscopic training, and how they view the use of video otoscopy as a teaching tool. Therefore, this study aimed to determine undergraduate (UG) audiology students’ perceived competence and confidence in conducting otoscopy following training by video otoscopic examination.

**Methods:**

A survey methodology with a cross sectional design was employed. An electronic questionnaire was distributed to all third and fourth year (senior) (*N* = 79) UG audiology students using Survey Monkey. Ethical approval and permission from relevant stakeholders were obtained. Data were analysed using both descriptive and inferential statistics.

**Results:**

60 % of the students felt competent in performing otoscopy, while 63.3 % felt less competent in interpreting otoscopic examination findings. 43.3 % felt they can confidently and competently identify outer ear pathologies. There was no association between the number of video otoscopic examinations performed and perceived competence or/and confidence. There was also no statistically significant relationship between year of study (e.g., third year versus fourth year) and perceived competence or/and confident (*p* = 0.7131). Almost all (97 %) students felt that video otoscopic training should continue to be part of the clinical training as it helped them enhance their skills in performing otoscopy.

**Conclusions:**

Current findings highlight the need to improve students’ practical training, incorporating pathologic ears into the curriculum. These findings also highlight the importance of supplementing practical training methodologies with changing technological advancements, particularly where tele-audiology opportunities may exist.

## Background

Otoscopic examination forms an important part of the standard audiological test battery for diagnosis of auditory disorders [1]; specifically, outer and middle ear disorders [2]. Yet, many students in hearing health programmes are not competent and confident in conducting otoscopic examinations [3, 4, 2]. The lack of competence and confidence in conducting otoscopic examinations may have clinical implications, leading to misdiagnosis of the outer and middle ear pathologies [5], which may be costly [6].

Literature has indicated that misdiagnosis of outer or/and middle ear pathologies may lead to a myriad of complications. These complications include, but are not limited to, permanent hearing loss, cognitive difficulties, and intracranial complications [7–9]. If these conditions are not treated timeously, they can affect the individuals’ communication and overall quality of life [10, 11]. To improve students’ abilities to identify early signs of outer and middle ear pathologies and reduce the likelihood of misdiagnosis, it is crucial to equip them with the necessary otoscopic examination skills. This is even more crucial in low-and-middle-income countries (LMICs) where hearing health resources are extremely limited and preventive ear and hearing care is crucial [12].

Several studies have been conducted to investigate the competence and confidence of students’ abilities to conduct, analyse and interpret otoscopic examinations [3, 13, 2]. These studies have used simulators to train and assess students’ abilities to analyse normal versus abnormal structures of the outer and middle ear; and significant improvement in performance was found following training. Although simulators may be useful in training students’ skills, simulators may not be available in resource limited countries, such as South Africa, due to their costs [14]. An alternative additional training method [4] is therefore required to ensure that students demonstrate competence in conducting, analysing, and interpreting otoscopic results before they graduate [15].

A video otoscope is a recent technological advancement and easy to use tool to examine the outer and middle ear structures [16, 17]. It typically uses a speculum that channels light, incorporates a video camera into the system, and allows for the transmission of images onto a screen [1], with visuals of typically the external auditory meatus (EAM) and the tympanic membrane (TM) [18]. Due to the added advantages illustrated above, Jaisinghani and colleagues [19] argue that video otoscopy can be used to effectively teach the anatomy and pathology of the EAM and the TM. Structural changes in the EAM and of the TM can provide valuable information about middle ear pathologies such as otitis media [20]. However, there is little research in South Africa in this area.

Audiology students in the department of Speech Pathology and Audiology at the University of the Witwatersrand were trained to conduct, analyse and interpret otoscopic findings using video otoscopy. Students were also instructed to capture additional video otoscopy, analyse and interpret results as part of their class assignment. However, it was not known if the video otoscopic training and class assignment had improved their competence and confidence in conducting, analysing, and interpreting otoscopic results. Therefore, the present study aimed to describe the audiology students’ perceived competence and confidence in conducting otoscopic examination following video otoscopic training.

### Research aim

The main aim of this study was to explore audiology students’ perceived competence and confidence in identifying outer and middle ear disorders following video otoscopic training, and to capture their views on video otoscopy as a training tool for ear visual inspection.

The specific objectives of the study were:

- To determine audiology students’ perceived competence in identifying outer and middle ear pathologies following video otoscopic training.
- To determine audiology students’ perceived confidence in identifying outer and middle ear pathologies following video otoscopic training.
- To describe audiology students’ views on video otoscopy as a training tool.

## Method

### Research design

This study employed a cross-sectional survey design [21]. This research design was chosen because it allowed the researchers to obtain information from a cohort of students at a given point in time. All procedures in this study adhered to the World Medical Association (WMA) Declaration of Helsinki (2013) ethical guidelines. Ethical approval was obtained from the University of the Witwatersrand’s Human Research Ethics committee (HREC) (non-medical) (Protocol Number: H19/09/49). Permission to conduct the study and to distribute the survey questionnaire to audiology students was obtained from the Head of the Audiology Department, and the University’s Registrar at the University of the Witwatersrand, Johannesburg, South Africa.

### Data collection method

Data collection was conducted using a self-administered online questionnaire. The questionnaire consisted of 17 Likert scale, multiple choice and closed and open ended questions, covering demographic information, perceived competence and confidence in performing otoscopic examination after video otoscopic training, as well as students’ recommendations regarding this form of training. The questionnaire consisted mainly of close-ended questions, with an option of ‘other’ which allowed audiology students to provide additional information. The questionnaire was sent to 3 audiologists to comment on the content and clarity of the questionnaire, and to provide appropriate suggestions for improvements [21]. The questionnaire was emailed together with a suggestion form. The questionnaire was then piloted on 4 audiology students who met the inclusion criteria, who did not form part of the main study. The final version of the questionnaire was uploaded onto the Survey Monkey and distributed to all third (n = 50) and fourth (n = 29) year students by the professional administrative (PA) staff members of the department of Speech Pathology and Audiology. Data collection took 6 months to complete (March 2020 to August 2020). The survey was distributed to students at least 4 times to increase the response rate.

### Participants

A non-probability purposive sampling was used to recruit and select participants [21]. Inclusion criteria included audiology students who underwent video otoscopic training. Participants in this study were 30 out of 79 UG audiology students (38 % response rate) at the University of the Witwatersrand, Johannesburg, South Africa. Prior to participating in this study, UG students had completed a 6-week revised course on anatomy, physiology, and pathology of the auditory system. This revision was then followed by a 3-week video otoscopic examination training. The training involved positioning of a patient, insertion of the speculum and channelling the light toward the tympanic membrane (TM) to obtain a clearer image, manoeuvring of the pinna when the TM cannot be visualized clearly, capturing of the image, saving of the image in a specific folder for this training, and analysing and interpreting the video otoscopic results. Students were also trained to identify any foreign objects such as occluded cerumen that may impede visualization of the tympanic membrane [18].

When training was completed, students were given a practical assignment to independently complete video otoscopic examination of 20 volunteer patients (40 ears each) and analyse the results. These volunteer patients were mainly university students from various training programmes or friends of students. Assignments were marked according to the following criteria: clarity of the captured images of the tympanic membrane, accuracy in interpreting all the tympanic membrane landmarks, and identification of pathologies if any existed. Constructive feedback was provided to students by the training facilitator.

### Data analysis

Descriptive statistics [22] using Statistical Package for Social Science (SPSS) version 25 were used to analyse the data. Frequency tables and graphs were used to summarize the data. Inferential statistics such as chi-squared were used to determine if there was any association between variables of interests such as year of study and competence/confidence, with p-value set at 0.05. A proportional test was also used to determine if there was any association between students’ performance during their assignment and perceived competence or/and confidence in conducting otoscopy.

## Results

**Fig. 1:**
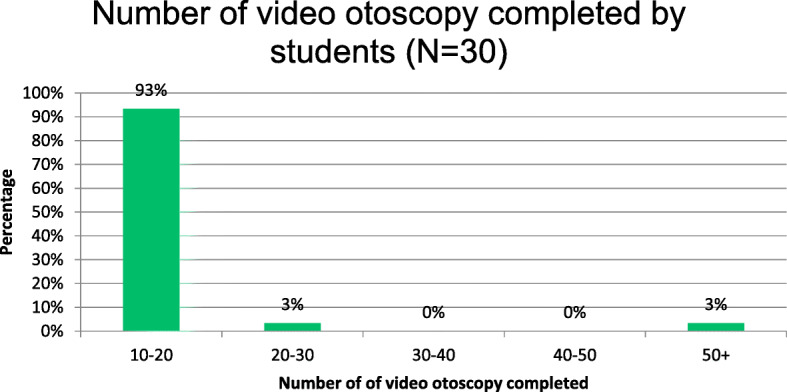
Video otoscopy completed by audiology students

Thirty audiology students participated in this study. All students who participated in the study were female audiology students who were in third and fourth year of study (where fourth year is the final year of study). As part of the practical assignment requirement for video otoscopy, majority of the audiology students (93 %) completed about 10 to 20 (40 ears) video otoscopic assessments, while only 3 % completed over 50 video otoscopic assessments (+ 100 ears). Figure 1 below illustrates the number of video otoscopic assessments completed by students.

Almost half (47 %) of the students indicated that they are somewhat confident, while 43 % felt very competent in the overall otoscopic examination. Half (50 %) of the students felt confident, while 60 % felt competent in performing otoscopic examination. 53 % of the students felt less confident while 63 % felt less competent in interpreting otoscopic examination. Further analysis showed that there was no statistical relationship between the number of video otoscopic examinations completed and perceived competence, X^2^ (6, *N* = 30) = 2.6374, *p* = 0.8528 or/and perceived confidence, X^2^ (8, *N* = 30) = 4.5153, *p* = 0.8079. There was no statistically significant relationship between year of study (e.g., third year) and perceived confident, X^2^ (4, *N* = 30) = 2.12, *p* = 0.7131. There was also no statistically significant relationship between year of study and perceived competence in conducting otoscopic examination, X^2^ (3, *N* = 30) = 4 3126, *p* = 0.2296.

**Fig. 2:**
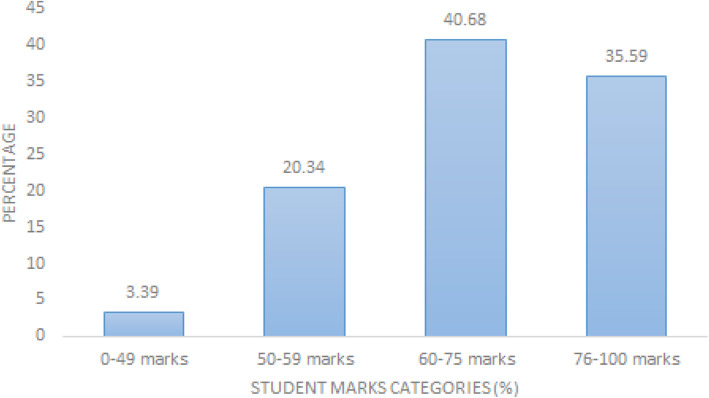
Student assignment marks categories

**Fig. 3:**
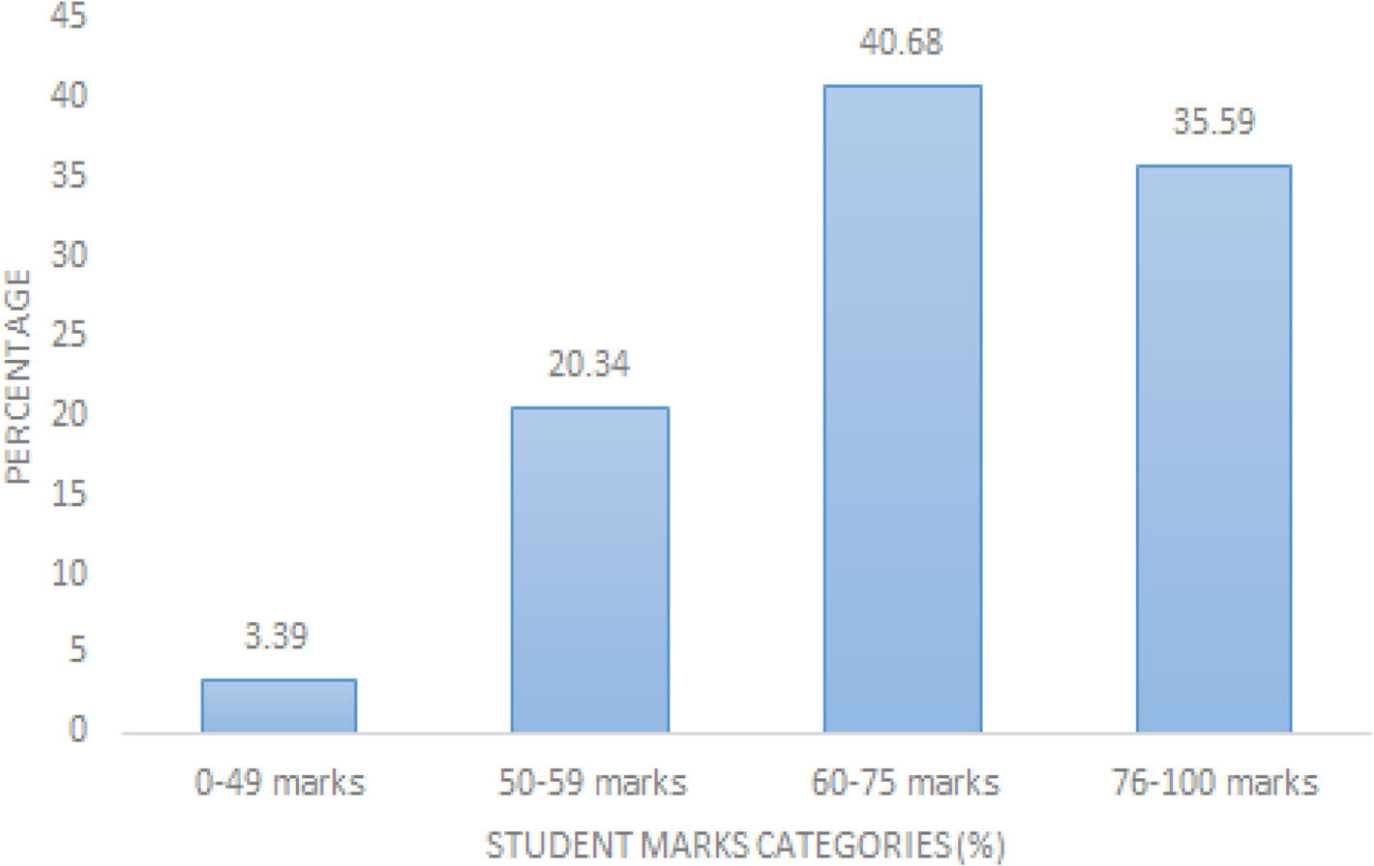
Students’ perception of competence in performing otoscopy

In order to determine whether there is any relationship between competence and students’ performance during their assignment, four levels of competence were created (see Figs. 2 and 3). A proportion test based on these categories and student marks was conducted. The somewhat competent and very competent proportion test indicates that these are not significantly different, while the not so competent and the extremely competent groups indicated some significant difference. This means that the proportion of those who indicated to be somewhat competent were not significantly different from the proportion of those who scored 50–59 %, similarly the proportion of those who indicated to be very competent were not significantly different from those who scored 60–74 %. However, since these data are independent of each other, correlation and the strength of correlation could not be established.

In terms of pathologies that students could identify using otoscopy, just under half of the students (43 %) felt that they can identify outer ear pathologies, while 37 % felt that they can identify both outer and middle ear pathologies.

Half of the students (50 %) felt that video otoscopic training helped them to become confident and competent in otoscopic examination, with the other half feeling that it had not helped them, and others feeling unsure. Participants who indicated that video otoscopic training helped them reported that this was due to the clear visualization of the tympanic membrane.

“You got a very clear and defined picture when using video otoscopy and it helped one become more comfortable at identifying the shape and bends of the ear canal, as well as the tympanic membrane” (participant 1).

“Tympanic membrane was more easily seen and interpretation for otoscopic results was much easier as well” (participant 2).

Participants who indicated that the video otoscopic training did not help them attributed this mainly to lack of time using the tool and machine dysfunction.

“Not having enough time with it because we had to share the device amongst students which takes time away from learning and mastering conducting and interpreting it” (Participant 3).

“The video otoscope did not function most of the time” (Participant 4).

Despite this, almost all students (97 %) indicated that video otoscopic training should continue to be part of the students’ clinical training.

## Discussion

Otoscopic examination is an important audiological procedure that helps in identifying early signs of both outer and middle ear pathologies [13]. In fact, research has indicated that otoscopic examination can identify middle ear pathologies much better than the conventional tympanometry with single probe tone [18] that is used by audiologists. It is therefore crucial that students are equipped with the necessary skills to be competent and confident in conducting otoscopic examination. This study therefore sought to determine if audiology students perceived themselves as competent and/or confident in conducting otoscopic examination following video otoscopic training.

Current findings suggest that approximately 50 % of the audiology students surveyed perceived themselves as competent and confident in conducting otoscopic examination, while the rest perceive themselves as less competent or/and confident. In terms of aspects of otoscopic examination that audiology students perceive themselves as less or more competent and confident in, majority of the students felt that they were less competent and confident in performing and interpreting otoscopic examination. The perceptions of audiology students in this study are inconsistent with previous studies that trained students [3, 13, 23]. Kaf and colleagues [13] found that majority of the audiology students (over 70 %) perceived themselves to be more confident after training; while You and colleagues [23] also found that the perception of medical students regarding confidence in otoscopic examination significantly improved following training.

The difference between the current findings and previous studies can be attributed to different training methods used. Kaf and colleagues [13] used Mannikin ears that reflected various statuses of the middle ear, while in the current study audiology students used University students mainly with normal middle ear function. The lack of exposure to various pathologies during training could have played a role in students’ perceptions. Students may be unsure on what they should be looking for when conducting otoscopic examination. Therefore, the findings of this study suggest a need for curriculum review for practical training of audiology students. Future training on otoscopic examination should consider incorporating patients with various middle ear pathologies.

In addition to video otoscopic training using volunteer patients, institution of higher learning, particularly in LMICs, should consider using simulations as part of clinical training as research suggests that simulations improve students’ confidence and competence [24, 25]. Wilson and colleagues [25] argue that simulations should support clinical training and not replace clinical training. Von Buchwald and colleagues [26] found that junior doctors scored low in identifying simulated middle ear pathologies. These authors argue that the difficulty in identifying pathologies may have been due to lack of case history accompanying the pathologic ears. Therefore, in LMICs, training programmes for audiology students should ensure inclusion of case history information as part of training to help students with the identification of outer and middle ear pathologies, and to make sure that the training is not in isolation.

Regarding specific pathologies that students can confidently and/or competently identify using otoscopy, 43 % of students felt that they can identify outer ear pathologies such as cerumen and foreign objects, while few indicated that they were able to identify outer and middle ear pathologies. These findings are also inconsistent with findings of a study conducted by Davies and colleagues [3] who found that 71 % of medical students perceived their confidence in diagnosing pathologies of the ear to have improved following training. Although audiology students are not expected to diagnose pathologies of the ear, it is within the scope of audiology for students to distinguish normal from abnormal outer and middle ear pathologies in order to be able to plan further testing as well as appropriate intervention and/or referral [13]. Therefore, it is crucial that students’ confidence and competence be improved for clinical best-practice purposes. Davies and colleagues [3] argue that lack of confidence in diagnosing ear pathologies ultimately affects graduates in clinical practice.

Despite the students’ lack of confidence and competence in identifying outer and middle ear, it was assuring that 97 % of the students felt that video otoscopy should continue to be part of their clinical training. This means students feel that video otoscopic training may improve their confidence and competence if done properly. In studies by Kaf and colleagues [13] and Davies and colleagues [3], training included images of pathologic ears to help students distinguish between normal and abnormal. Therefore, further research in this area should ensure that pathologic ears are included in the training.

There is an increasing effort to develop mobile applications to facilitate identification and management of auditory and otolaryngological pathologies, including outer and middle ear pathologies [27]. Given the documented demand versus capacity challenge in LMICs, mobile applications can be used to help with the training of students to identify outer and middle ear pathologies, where instructors other than those physically available in local training institutions can be utilised. Bhavana and colleagues [28] found a higher sensitivity and specificity of smartphone otoscopy in identifying outer and middle ear pathologies when compared with oto-endoscopy. Myburgh and colleagues [29] developed an automated cloud-based smartphone system which uses image analysis to diagnose outer and middle ear pathologies. These mobile applications are crucial in LMICs and can be used to teach audiology students to be able to identify early signs of outer and middle ear pathologies; and can also facilitate peer and/or supervisor support though asynchronous tele-audiology modalities.

Given that the COVID-19 pandemic has severely affected the traditional face-to-face audiological service provision [30], including student training in institutions of higher learning, Khoza-Shangase and colleagues [31] emphasise the need for innovative models of service delivery and student training to ensure the continuity of care and student training during and beyond this pandemic. Telepractice and teletraining have been at the forefront to ensure continuity of care and student training. Bhavana and colleagues [28] argue that smartphone otoscopy can be used as a teaching tool within the telemedicine approach, especially in LMICs. Sebothoma and Khoza-Shangase [32] and Binol and colleagues [33] indicate that video otoscopic images of the tympanic membrane can be used within the telemedicine approach. Given the high sensitivity and specificity of video/digital otoscopy, this has important training and practice implications, particularly during the COVID-19 pandemic.

While the current study provides important evidence about the use of video otoscopy as a teaching tool for outer and middle ear pathologies, it is worth noting that at the time of this study, there was 100 % female audiology students from third to fourth year at the University where the study was conducted. Although this gender profile may be slightly different in other universities, the predominant number of females in the cohort of students is not surprising given that the gender profile of the South African audiologists and speech therapists registered with the Health Professional Council of South Africa (HPCSA) is primarily (95 %) female [34]. These professions are highly feminised in South Africa, with efforts to transform the gender, linguistic and racial demographic profile afoot in the country. Despite this, it is not believed that this profile had an impact in the current findings. In addition, the sample size for this study was very small, with a response rate of 38 % which is significantly lower than the ideal 60 %; and this certainly is believed to have had an impact on the current findings; and influences their generalizability. The impact of COVID-19 pandemic on data collection for this study highlighted the importance of enhancing tele-training and tele-data collection strategies in any training programme.

## Conclusions

Audiology students in this study were somewhat confident and competent in conducting otoscopic examination following video otoscopic training. Further research is needed to determine if perception of students correlates to actual hands-on improvement of their otoscopic skills. This study also highlights the need for improving practical curriculum training of students in this area. Incorporating pathologic ears may be helpful in improving audiology students’ confidence and competent in identifying outer and middle ear pathologies. This may help students to make appropriate referrals for medical management. Furthermore, this study also highlighted the need to supplement practical training methodologies with changing technological advancements; and enhancing tele-training and tele-data collection strategies going forward.

## Data Availability

All data generated or analysed during this study are included in this published article.

## Abbreviations

COVID-19: Coronavirus disease 19
EAM: External auditory meatus
HREC: Human Research Ethics Committee
LMICs: Low- and middle-income countries
SPSS: Statistical Package for Social Science
PA: Professional administrators
TM: Tympanic membrane
UG: Undergraduate
